# An implementation-focused process evaluation of an incentive intervention effectiveness trial in substance use disorders clinics at two Veterans Health Administration medical centers

**DOI:** 10.1186/1940-0640-9-12

**Published:** 2014-07-09

**Authors:** Hildi J Hagedorn, Cheryl B Stetler, Ann Bangerter, Siamak Noorbaloochi, Maxine L Stitzer, Daniel Kivlahan

**Affiliations:** 1 Veterans Health Administration Substance Use Disorder Quality Enhancement Research Initiative, Minneapolis VA Healthcare System, One Veterans Drive, Minneapolis, MN 55417, USA; 2 Veterans Health Administration Health Services Research and Development Center of Excellence, Minneapolis VA Healthcare System, Minneapolis, MN 55417, USA; 3 University of Minnesota, Minneapolis, MN 55455, USA; 4 Health Services Department, Boston University School of Public Health; and Independent Consultant, Amherst, MA 01002, USA; 5 Johns Hopkins University School of Medicine, Baltimore, MD 21224, USA; 6 Center of Excellence in Substance Abuse Treatment and Education, VA Puget Sound Healthcare System, Seattle, WA 98108, USA; 7 University of Washington School of Medicine, Seattle, WA 98185, USA

**Keywords:** Substance use disorders treatment, Abstinence incentive intervention, Implementation, Process evaluation, Hybrid design

## Abstract

**Background:**

One of the pressing concerns in health care today is the slow rate at which promising interventions, supported by research evidence, move into clinical practice. One potential way to speed this process is to conduct hybrid studies that simultaneously combine the collection of effectiveness and implementation relevant data. This paper presents implementation relevant data collected during a randomized effectiveness trial of an abstinence incentive intervention conducted in substance use disorders treatment clinics at two Veterans Health Administration (VHA) medical centers.

**Methods:**

Participants included patients entering substance use disorders treatment with diagnoses of alcohol dependence and/or stimulant dependence that enrolled in the randomized trial, were assigned to the intervention arm, and completed a post intervention survey (n = 147). All staff and leadership from the participating clinics were eligible to participate. A descriptive process evaluation was used, focused on participant perceptions and contextual/feasibility issues. Data collection was guided by the RE-AIM and PARIHS implementation frameworks. Data collection methods included chart review, intervention cost tracking, patient and staff surveys, and qualitative interviews with staff and administrators.

**Results:**

Results indicated that patients, staff and administrators held generally positive attitudes toward the incentive intervention. However, staff and administrators identified substantial barriers to routine implementation. Despite the documented low cost and modest staff time required for implementation of the intervention, securing funding for the incentives and freeing up any staff time for intervention administration were identified as primary barriers.

**Conclusions:**

Recommendations to facilitate implementation are presented. Recommendations include: 1) solicit explicit support from the highest levels of the organization through, for example, performance measures or clinical practice guideline recommendations; 2) adopt the intervention incrementally starting within a specific treatment track or clinic to reduce staff and funding burden until local evidence of effectiveness and feasibility is available to support spread; and 3) educate staff about the process, goals, and value/effectiveness of the intervention and engage them in implementation planning from the start to enhance investment in the intervention.

## Introduction

One of the most pressing concerns in healthcare today is the slow rate at which promising clinical interventions, supported by research evidence, move into clinical practice [1-6]. While effectiveness trials are a step in the research pipeline designed to determine whether promising clinical interventions work when tested in “real world” settings, the results of such trials generally do not provide clinicians, administrators, or quality improvement specialists with the information that they will need to successfully implement the new practice in their clinic or hospital [7-10]. This is the role of implementation trials which attempt to develop successful strategies for implementing new, effective practices, which then can be disseminated/spread to other clinical settings [8,11].

Unfortunately, the timeline from a promising efficacy trial, through effectiveness and implementation trials, and finally to dissemination can be measured in multiple years if not decades and adds to the slow rate of routine uptake of promising clinical interventions [4,12-14]. Methods to speed this process are needed. One recently proposed method is to integrate effectiveness and implementation study methods using a hybrid design [15]. Curran and colleagues propose three hybrid types varying in the degree to which they focus on effectiveness data versus implementation data (See Table 1). The Hybrid Type I encourages researchers to initiate the study of implementation as early as possible in a program of research; more specifically, this design focuses on integrating implementation research into efforts primarily focused on collecting effectiveness data. As described by Curran and colleagues, the additional incorporation of process evaluation methods into an effectiveness trial design can help explain effectiveness results while at the same time efficiently informing future implementation efforts. In contrast to process evaluation, as defined in this study as part of a Hybrid I design, formative evaluation [9] is used in a Hybrid II or III design to gather information for monitoring and **actively** enhancing implementation during the course of the implementation-focused aspect of the study.

**Table 1:** Typology of hybrid designs*

**Research questions**	**Hybrid type 1**	**Hybrid type 2**	**Hybrid type 3**
**Primary**	**Effectiveness:** Will the clinical treatment work in this setting/with these patients?	**Effectiveness:** Will the clinical treatment work in this setting/with these patients?	**Implementation:** Which method works better in facilitating implementation of the clinical treatment?
		AND	
		**Implementation:** Does the implementation method show promise?	
**Secondary**	**Implementation:** What are the potential barriers/facilitators to the treatment’s implementation?		**Effectiveness:** Is a clinical treatment effective in this setting/with these patients?

*From Curran, Bauer, Mittman, Pyne & Stetler, [15].

Traditionally, process evaluations are used when testing complex clinical interventions and involve collecting data from patients and providers to assess issues such as whether the clinical intervention was delivered as designed, whether the patients received the planned “dose” of the clinical intervention, and whether providers maintained fidelity to the clinical intervention protocol [16-18]. Implementation questions in Hybrid Type I studies instead focus more prominently on barriers and facilitators to uptake/adoption efforts, feasibility of the clinical intervention, acceptability of the clinical intervention to providers and patients, and on identifying tools and training that would assist with high fidelity implementation. Therefore, information is collected not only from patients and providers but from clinical and organizational leaders that would be responsible for guiding implementation of the clinical intervention.

Process evaluations frequently involve mixed-methods, combining data collected through qualitative methods such as interviews, focus groups, and field notes with data collected through quantitative methods such as surveys and administrative data. While there has been a call for hybrid studies integrating quantitative effectiveness data with quantitative and qualitative implementation-focused process evaluation data, there are few published protocols documenting the design, execution, and results of such trials. This paper describes an implementation-focused process evaluation that was tied to a randomized, controlled effectiveness trial of an abstinence incentive intervention for substance use disorders [19]. The current paper reports how the process evaluation was developed and carried out, results of the evaluation, and discussion of how these results may inform future implementation efforts.

## Methods

### Participants

This study was conducted at two VHA medical centers. At one medical center, participants were recruited from the outpatient substance use disorders treatment clinic and a dual diagnosis partial hospitalization clinic. At the second medical center, participants were recruited from the outpatient substance use disorders treatment clinic. As approved by the institutional review boards at each facility, patient participants completed written informed consent and staff participants completed verbal informed consent. Patients were eligible to participate if they were beginning a new treatment episode for alcohol dependence and/or stimulant dependence. All clinical staff and clinic leaders were eligible to participate. Clinical staff was recruited through announcements at clinical staff meetings and emails. Clinic leaders were recruited through email and telephone contacts. During the study, patients were randomized to receive usual care substance use disorders treatment with or without financial abstinence incentives. All participants were scheduled to submit alcohol breath and drug urine screens twice per week for eight weeks. Those in the incentive condition could draw from a prize bowl when they submitted negative breathalyzer and urine screens. The prize bowl contained slips that either said “Good Job” but had no monetary value or were worth $1, $20, or $80 vouchers, which could be redeemed at a VHA cafeteria or gift shop. The number of drawings earned increased with each consecutive week of abstinence.

### Process evaluation plan

The initial step in development of the process evaluation plan was the selection of an implementation framework that would guide the questions asked. For the purpose of this study, our team selected the RE-AIM framework [20-22] as the primary guide supplemented with elements from the Promoting Action on Research Implementation in Health Services (PARIHS) framework [23-25]. RE-AIM is an acronym for *reach, effectiveness, adoption, implementation* and *maintenance.* Briefly, *Reach* refers to the percentage of eligible patients that agree to the clinical intervention. *Effectiveness* evaluates whether the clinical intervention achieved the desired patient outcomes. *Adoption* refers to the decision of the leadership and staff at the targeted settings to implement the clinical intervention. *Implementation* refers to whether the clinical intervention is implemented with high fidelity and consistency. Implementation also includes an assessment of the cost of the intervention. Finally, *Maintenance* refers to whether the clinical intervention is sustained after implementation support is withdrawn. The framework proposes that each of these elements is essential to consider when planning an implementation strategy or an implementation evaluation*.* According to Kessler and colleagues [26], RE-AIM has been used to “plan, evaluate and review a variety of health promotion and disease management interventions” and “over 150 published studies… state they are using the RE-AIM model.” However, many of these studies did not include a comprehensive evaluation of all framework elements. See Kessler and colleagues’ paper [26] on employing the RE-AIM framework for an excellent description of general questions prompted by each element of the model.

Guided by the framework, we developed a series of questions that would provide valuable information for future implementation efforts. (See Table 2, which also includes measurement tools discussed below.) Because our purpose was to collect information during an effectiveness trial to inform future implementation rather than to evaluate an actual implementation effort, our questions are slightly different from those recommended by Kessler [26].

**Table 2:** Implementation framework elements guide process evaluation questions and tools

**Element**	**Questions**	**Data sources**	**Tools**
**RE-AIM**			
Reach	What percentage of patients approached agree to participate in the intervention?	Recruitment rates.	Patient screening database.
	Do those that agree to participate differ systematically from those that do not?	Demographics of those agreeing vs. those refusing participation.	Chart review of administrative data.
	What do patients like and dislike about the intervention?	Perceptions of patients.	Post-intervention patient surveys.
Effectiveness^a^	What is the effect of the intervention on patient outcomes?	Main study outcomes comparing control to intervention patients.	Rates of negative urine screens.
			Study retention.
Adoption	What are the greatest barriers to adopting the intervention?	Perceptions of staff and leadership.	Research team observational log.
			Post-intervention staff interviews.
	What supports will need to be in place for clinics to adopt the intervention?		Post-intervention leadership interviews.
Implementation	What supports need to be in place to ensure consistent delivery of the intervention?	Perceptions of staff and leadership.	Post-intervention staff interviews.
			Post-intervention leadership interviews.
	What tools will be needed to deliver the intervention consistently?	Perceptions of research staff	Training protocols for research staff.
	What does the intervention cost?	Cost data.	Records of incentives awarded, costs of intervention supplies and staff time required.
Maintenance	What resources will be needed to maintain the intervention in the long run?	Perceptions of leadership.	Research team observational log.
			Post-intervention leadership interviews.
	What adaptations will need to be made to integrate the intervention into regular practice?		
**PARIHS**			
Evidence	What are staff perceptions of the evidence supporting the intervention?	Perceptions of staff and leadership.	Organizational Readiness to Change Assessment (ORCA) Evidence Scales
	What are their attitudes toward the intervention?		
	Does the intervention fit with their current clinical practice?		
	Does the intervention meet a perceived need of their patients?		
Context	What are the characteristics of the culture in the clinic?	Organizational readiness measure collected from staff and leadership.	Organizational Readiness to Change Assessment (ORCA) Context Scales
	What are the characteristics of the leadership of the clinic?		
	What resources are available to the clinic?		

^a^Effectiveness outcomes are reported elsewhere [19].

The PARIHS framework proposes that successful implementation is a function of *evidence, context* and *facilitation*. For our purposes, we chose to evaluate the elements of *evidence* and *context* as follows:

*Evidence:* In the PARIHS framework, this element refers to the strength and extent of evidence for clinical practice changes and includes research evidence, evidence from clinical practice, perceived patient needs and preferences, and local data/information. The RE-AIM framework lacks a component specifically addressing clinicians’ assessments of the evidence supporting an innovation.

*Context:* In the PARIHS framework, this element refers to “the environment or setting in which people receive healthcare services” or, in the context of getting research evidence into practice, ‘the environment or setting in which the proposed change is to be implemented” [23]. Given the importance of context in a number of implementation frameworks e.g., [27-29] and the fact that RE-AIM does not specifically address context, we felt that assessing this element would add important information to our process evaluation. Specifically, it could enhance our interpretations of setting-related barriers and facilitators to this innovation.

PARIHS’s *facilitation* element is defined as an appointed role wherein an individual helps and enables others to change [23]. Evaluation of *facilitation* was not felt to be relevant to this study as the research staff administered the study’s clinical intervention and were not providing such help or support to assist clinical staff with real-time implementation. However, information gained from assessing the RE-AIM framework, as well as PARIHS’ *evidence* and *context* elements, was expected to provide sufficient information to inform development of future facilitation strategies.

### Data collection tools

Data tools included pre-existing scales, surveys and interviews designed specifically for this study, observational notes, data forms completed by research staff, and chart reviews to collect administrative data (Table 2).

#### Chart review data

In order to track *Reach* of the intervention, a screening database was maintained which contained basic demographic and diagnostic information on every patient that was approached regarding participation in the study. This information was used to determine the percentage of patients approached that agreed to participate and to compare basic demographic and diagnostic characteristics of those agreeing and declining participation.

#### Cost records

One consideration in regards to *Implementation* of a new intervention is the cost associated with the intervention. A standardized computer data entry screen was used to document the amount of time required for each participant intervention appointment and the monetary value of any vouchers earned. Staff time was converted into costs using three different wage rates plus a 30% fringe rate. Average wage rates were calculated for: 1) licensed practical nurses, which clinic leadership indicated would be the most likely staff position to administer the intervention if one person were assigned to administer it to all patients, 2) the actual research staff that administered the intervention for the study (Bachelor’s level research assistants), and 3) licensed clinical social workers, which clinic leadership indicated would be the most likely staff position to administer the intervention if each case manager were assigned to administer it to their own patients.

#### Patient surveys

To collect information that could improve the *Reach* of the incentive intervention, participating patients completed post-intervention surveys at the end of the eight week intervention period. The survey queried patients about what they liked and did not like about the intervention, any interactions they had with their clinical treatment team related to their participation in the intervention, and their thoughts on potential modifications to the incentive intervention.

#### The Organizational Readiness to Change Assessment (ORCA)

The ORCA is a 77-item scale designed to measure elements of the PARIHS framework [30,31]. Items pertaining to the *evidence* and *context* elements were administered.

The *evidence* scale both assesses the respondents’ opinions regarding support for the proposed intervention based on research evidence, clinical experience and patient preferences, as well as their perception of colleagues’ related support. Because preliminary discussions with staff indicated that incentive interventions can be controversial, we added an additional eight items to assess personal opinions and intentions regarding incentive interventions (see Table 3). The *context* scale contains six subscales: leadership culture, staff culture, leadership practices, measurement (e.g., leadership feedback), readiness to change among opinion leaders and resources to support general practice changes.

**Table 3:** Items added to organizational readiness to change scale to assess providers personal preferences and intentions

**Construct**	**Item**
Preferences	• If monetary rewards can help patients to achieve abstinence, I support it.
	• Giving patients monetary rewards for staying abstinent or attending treatment is wrong. (reverse coded)
	• Patients who really want to stay abstinent shouldn’t need monetary rewards. (reverse coded)
	• We need to use every possible tool, including monetary rewards, to help patients succeed in treatment.
	• Giving patients monetary rewards for abstinence or treatment attendance is an inappropriate use of VHA funds. (reverse coded)
	• Giving patients monetary rewards will recognize their achievements and give them a sense of accomplishment.
Intentions	• I would like to see the use of incentive interventions in this clinic.
	• There are specific plans in place to implement an incentive intervention in this clinic.

ORCA surveys were handed out at clinical staff team meetings after all patient participants had completed the intervention phase of the trial. No identifying information was collected so that completion of the assessment was voluntary and anonymous.

#### Staff interviews

To collect information on *Adoption, Implementation* and *Maintenance*, after all patient participants had completed the intervention phase of the trial, staff members that agreed to be interviewed were asked their opinions regarding the intervention and how it impacted the clinic, their interactions with their patients, and their workload. They were also asked about barriers and facilitators to maintaining the intervention as part of standard clinical care. No identifying information was collected during the interviews other than the staff members’ role in the clinic.

#### Leadership interviews

To collect information on *Adoption, Implementation* and *Maintenance,* after all patient participants had completed the intervention phase of the trial, clinic leaders were asked about their plans to continue the incentive intervention in their clinic, perceived incentives for maintaining the intervention, barriers and facilitators to maintaining the interventions, and types of support that would be most helpful in adopting and maintaining the intervention.

#### Research staff observational log

To supplement information regarding *Adoption, Implementation* and *Maintenance,* a shared research staff observational log was created to document interactions with clinical staff and patients that provided information on their expressed attitudes toward the intervention and their comments on perceived barriers and facilitators to adoption, implementation, and maintenance of the intervention. The idea behind the log was that opinions might surface during day-to-day interactions with research staff that might not be readily reported when utilizing more formal data collection methods such as surveys and interviews.

### Data analysis

Descriptive quantitative results are reported in percentages. Chi-square tests, and Z or t-tests were used to compare distributions, proportions and means of different subgroups. Qualitative information from open-ended survey questions, interviews, and research log entries were transcribed and analyzed using common coding techniques for qualitative data [32]. The principal investigator (HH) and the research coordinator (E. Marut) independently reviewed the transcripts and developed initial coding lists. A deductive analysis approach was used during the development of the initial coding list to focus codes on pre-determined domains of interest. This included attitudes toward the intervention, barriers to and facilitators of implementation, and recommendations for improvement of the intervention. Following development and refinement of the initial coding list, each transcript was coded, with additional inductive codes added that identified important themes not represented by the pre-determined domains. Consensus meetings were held for review of consistency in coding. Inconsistencies were resolved through mutual discussion and agreement.

## Results

### Patient screening data

**Figure 1:**
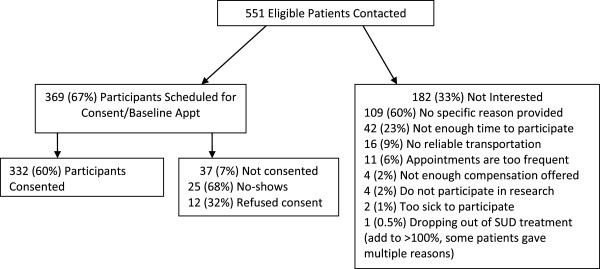
Participant recruitment.

Figure 1 provides recruitment information including percent of contacted patients that participated and declined and reasons for declining. Table 4 provides demographics for those that declined participation and those that agreed to a baseline appointment. African Americans and patients with stimulant dependence diagnoses agreed to participate at higher rates than patients in other demographic and diagnostic categories.

**Table 4:** Demographic characteristics of patients that declined participation and patients that scheduled baseline appointment

**Characteristic**	**Declined participation**	**Scheduled**
	**N = 182 (33.03%)**	**N = 369 (66.97%)**
Male	178 (97.80%)	361 (97.83%)
Female	4 (2.20%)	8 (2.17%)
African American	34 (18.68%)	136 (36.68%)
White	124 (68.13%)	194 (52.57%)
Other non-white	4 (2.20%)	10 (2.71%)
Age in years, median	52.5	50
Psychiatric Diagnoses		
Depression	59 (32.42%)	131 (35.50%)
PTSD	29 (15.93%)	46 (12.47%)
Other anxiety, mood, or psychotic diagnosis	37 (20.33%)	53 (14.36%)
Substance Dependence Diagnoses		
Alcohol Dependence	172 (94.51%)	307 (83.20%)
Stimulant Dependence	32 (17.58%)	156 (42.28%)
Other Substance Dependence	18 (9.90%)	33 (8.94%)

### Costs

Table 5 presents cost information. Most substance use disorders clinics have a breathalyzer unit available so the cost of the unit was not included as a cost of the intervention. However, if a clinic needs to purchase a breathalyzer, one can be purchased for less than $500. The system to track appointment length indicated that mean appointment length was 7.0 minutes (SD = 4.5). Costs were assessed based on 15 minute appointments because the minimum appointment scheduling time in the VHA is 15 minutes and 15 minutes per appointment would have allowed sufficient time for 96% of all appointments. Staff costs were multiplied by the 16 scheduled appointments rather than the number of appointments actually attended. This accounted for staff time spent documenting no-shows and contacting patients to reschedule.

**Table 5:** Costs of the intervention

**Expense item**	**Cost (Mean)**
Incentive Vouchers	$103.07
Rapid Urine Test Cups	$60.90
($5.25/cup × 11.6^a^)	
Breathalyzer Mouthpieces	$2.78
($0.24 × 11.6)	
Staff Costs	LPN^b^ = $102.72
(16 appointments × 15 minutes each)	BA^c^ = $125.60
	LCSW^d^ = $152.00
Total Cost per Patient	$266.93 - $316.21

^a^11.6 = mean number of intervention appointments attended.

^b^Licensed Practical Nurse: $41,101 base + 30% fringe.

^c^Bachelor’s level research assistant: $50,275 base + 30% fringe.

^d^Licensed Clinical Social Worker: $60,827 base + 30% fringe.

### Patient surveys

The post-intervention survey was completed by 147 of the 165 patients (89%) that were assigned to the incentive intervention. When asked what they liked about participating in the intervention, 94% of participants gave a response. When broadly categorized, the top response categories were “liked earning the rewards” (44.3%), “the intervention supported abstinence” (37.0%), and “liked working with the research staff” (22.4%). One participant summarized her experience in the following way:

“It was very positive. The program doesn’t dwell on your short-comings. It is focused on positives with the incentives. It helped me to stay sober and helped me to commit to not drinking. I really liked it.”

When asked what they did not like about participating in the intervention, 78% of participants stated “nothing.” The most frequent category, reported by 10% of participants, were complaints about the reward system, e.g., the rewards were not big enough. Participants stated that:

“I didn’t like the reward scale, the odds of not winning.”

“Those “Good Job” things, I didn’t like picking those.”

“Dang it, (name of other participant) won $80 and not me!”

Other responses included “the appointments were inconvenient” (8%) and “did not like the testing procedures” (3%).

When asked to rate the impact of the incentive intervention on their personal treatment outcomes, 81 (55.1%) participants rated the impact as highly positive, 47 (32.0%) participants rated the impact as somewhat positive and 19 (12.9%) participants rated the incentives as having no impact on their treatment outcomes. None of the participants rated the impact of the incentives on their personal treatment outcomes as either somewhat or highly negative.

Only 22.8% of patients reported that someone from the clinical team had discussed their intervention participation with them. Of these conversations, the majority were neutral (63.0%) and involved a clinical staff person either mentioning the opportunity to be involved in the study or asking how participation was going. The remainder of the conversations were positive (37.0%) with the clinical team member indicating that it was a good thing for the patient’s recovery to be involved in the intervention. There were no reports from patients of clinical staff making negative comments about their intervention participation. There were only two instances where a patient reported that a clinical team member discussed specific urine and breathalyzer screen results from the intervention with the patient.

Participants indicated that the intervention would have been more effective if it had been longer (greater than 8 weeks), if the prizes available had been bigger, and if it offered a larger number of prizes and more large and jumbo prizes. The participants indicated that the intervention would have been less effective if it had been shorter (less than 8 weeks), if the appointments had been less frequent (less than 2 times per week), and if the intervention had taken place in a group setting where patients would know each other’s test results and reward earnings.

### Organizational readiness to change assessment

The ORCA was completed by 13 staff members from each of the two participating substance use disorders clinics (Total N = 26/39, 67% response). At the first site, respondents included five social workers, three nurses, two psychiatrists, one psychologist, and two staff members that did not record their role in the clinic. At the second site, respondents included five social workers, one nurse, three psychiatrists, three psychologists, and one staff member that did not record his/her role in the clinic.

Staff at both sites expressed agreement with the evidence supporting incentive interventions. On a scale of 1 to 5, with 5 representing strong agreement, the mean evidence scale rating was 3.7 for site one and 3.9 for site two. Mean scores on the three evidence subscales (research evidence, clinical experience, and patient preferences) were all above 3.7 for both sites. None of the differences between sites were significant. On the six items related to personal opinions regarding incentive interventions, site one had a significantly lower rating (M = 3.5) compared to site two (M = 4.2; p = .01) although site one was still in the supportive range (> 3). In regards to the intentions questions, on the item asking whether they would like to see an incentive intervention continue in their clinic, site one had a significantly lower rating (M = 3.8) than site two (M = 4.5; p = .027) but both sites were in the supportive range. In looking at individual scores, site one had three individuals that had clearly negative opinions regarding the incentive intervention which pulled down the overall score for the clinic despite the majority of staff expressing support. When asked if there were any specific plans in place to continue an incentive intervention in the clinic, the mean rating for both sites was 2.8 indicating that while they were supportive of the intervention, they were unaware of any clear plans in place to sustain it following the research study.

Staff at both sites also indicated that their sites had contexts that were supportive of innovation in general. The mean context scale rating was 3.6 for site one and 3.3 for site two. Mean scores on five of the six context subscales (leadership culture, staff culture, leadership practices, measurement, and opinion leaders) were all above 3.1 for both sites. None of the differences between sites were significant. The only context subscale to have negative mean ratings from both sites was the resource subscale indicating that staff at both sites did not feel that they had the financial resources, training, facilities or staffing necessary to adequately support implementation of innovations. Site two had a significantly lower mean rating on resources (M = 2.0) than site one (M = 2.9; p = .01).

### Staff interviews

Seven interviews were conducted with staff from site one and two were conducted with staff from site two. The professional roles of those interviewed included two nurses, two social workers, one psychologist, one addiction therapist, and three whose roles were not identified.

When staff were asked what they liked about having the incentive intervention in their clinic, the most common themes to emerge, mentioned by three or more respondents, were that the intervention motivated patients to continue treatment, the intervention provided the patients with tangible rewards for success, the intervention provided the patients with additional support, the intervention provided the staff with objective outcome data, and that the intervention was a positive/enjoyable experience for patients. For example, one staff member summed up her experience with the intervention in the following way:

“I liked it because it motivated the clients. There was a possibility for them to win something. Also, praise goes a long way. They come to (name of treatment program) beaten down and the incentives provided them with positive reinforcement.”

When staff was asked what they did not like about having the incentive intervention in their clinic, six of nine responded nothing. Two staff reported a concern that the intervention focused patients on external rather than internal motivation for change as exemplified by the following quote:

“Sometimes it seemed that the patients enrolled in the intervention were more superficial in their treatment. They had to stay sober for the rewards and money, not for deeper reasons.”

Two staff also reported a concern that the intervention could trigger gambling behavior.

When asked whether the intervention had any impact on clinic functioning or atmosphere, the most common theme, mentioned by five respondents, was that the intervention provided an additional opportunity to support patients’ recovery, as exemplified in the following quote:

“The intervention added to the overall goal of working together as a team to help the patients. I think the patients got the sense that, as a clinic, we were committed to helping them to get and stay sober. The incentive study helped with that.”

None indicated a negative impact of the intervention. When asked whether the study intervention had any impact on individual respondents’ workloads, six of nine respondents stated that there was no impact. Those that reported an impact reported co-signing incentive intervention appointment notes and having more contact with patients during the aftercare phase of treatment. All three agreed that this increased interaction improved treatment for the patients. As one staff member described it:

“There were a lot of notes to cosign, although it was always optional. I like to have the information about who was showing up for intervention appointments and who was testing positive and negative.”

When asked if the intervention influenced their interactions with their patients, five of nine respondents indicated that it did not. Those that did indicate an influence reported talking with their patients about their study earnings and adding ongoing participation in the intervention to patients’ aftercare recovery plans.

When asked if they would like to see the intervention continue in the clinic after the research study ended, seven of the nine respondents said yes. The remaining two stated that their opinion would depend on the results of the study. When asked about barriers to adopting the intervention as part of standard clinical treatment, the most common theme, cited by six respondents, was cost. An additional barrier mentioned by three respondents was allocating staff time to administer the intervention. As summed up by one staff person:

“The time commitment would be a barrier. You need staff that would be in charge of this. The expense would also be a barrier. I see the lab costs as being the most expensive cost. Sending confirmations to the lab is very expensive but I think it’s really important that we do that.”

When asked what would make it easier to adopt the intervention as part of standard clinical treatment, themes reported by three or more respondents were dedicated staff and soliciting buy-in from all staff in the clinic prior to implementation to decrease resistance. For example, one staff person stated:

“Everybody needs to be on the same team. There needs to be staff buy-in from everyone involved. Another thing is that it should not be considered a secondary part of treatment. It should be an integral part.”

### Leadership interviews

All three leaders of the participating clinics indicated that they were interested in implementing an incentive intervention but that it was not currently feasible due to significant barriers. In the outpatient substance use disorders clinics, the main perceived barriers were as follows: staff were already stretched beyond capacity, the need to either gain additional staff or reallocate staff time from other priorities, and the need to identify a funding source for both the incentives and the rapid urine test cups. As one of the leaders put it:

“The biggest barrier is that I would have to pull staff from other tasks in order to do it. There are only so many staff members and something else would have to give.”

In the dual diagnosis partial hospitalization clinic, the main barrier cited was that their program was primarily group based and therefore, they would need expert consultation to determine the best way to incorporate the intervention into the group setting. Staff time was also a concern for this clinic.

All of the leaders indicated that there were significant incentives for pursing implementation. The leaders in the substance use disorder treatment clinics noted that the intervention appeared to improve treatment retention and that this improved their clinics’ functioning on a VHA substance use disorders treatment continuity of care performance measure that was monitored by national leadership as demonstrated by the following quote:

“For me, the continuity of care is a big incentive. It (the intervention) keeps the patients engaged in treatment and coming in for appointments.”

The leader of the dual diagnosis clinic noted that incentive interventions are an empirically supported treatment and that empirically supported treatments generally, and incentive interventions specifically, were strongly supported by national leadership. When asked what types of assistance would be most beneficial to support implementation, suggestions included educating staff and facility leadership regarding the evidence for incentive interventions and expert consultation in designing an incentive intervention that would fit into the current practices of the clinic.

### Research observational log

Research staff recorded over 150 contacts with clinical staff in the log. The vast majority of recorded contacts were positive. Clinical staff demonstrated support for the intervention both through assisting the research staff with recruitment of participants and with maintaining contact with participants during enrollment in the study. Clinical staff comments indicated the intervention was useful to their work with patients by allowing regular monitoring of urine screen results and keeping patients connected to aftercare. When one of the research assistants approached a staff member about recruitment of a specific patient, the staff member spontaneously replied:

“I really appreciate how the study keeps the patients coming back to the clinic. It helps keep them connected to aftercare after they complete their intensive program. It gives them another long term connection and support.”

The research log also prompted the research staff to identify and act on suggestions from clinicians to enhance clinician involvement with the intervention. For example, staff at both facilities requested that they receive regular updates on participants’ progress in the incentive intervention. Research staff worked with the clinicians to identify the best way to achieve this goal at each facility. A second suggestion that came from clinical staff was that the patient’s case manager should be invited to the final intervention appointment in order to celebrate this achievement with their patient; however when this was offered, staff attended infrequently.

As stated above, the vast majority of recorded interactions were positive but there were barriers and negative responses to the intervention recorded as well. The major barrier to integrating the intervention into clinic processes at one facility was that the clinicians did not want to rely on the rapid urine test cups for clinical testing of substance use. They preferred to send samples directly to the laboratory. This created a great inconvenience for patients who were often asked to submit two urine samples (one for research and one for clinical purposes) within a very short time frame. During the course of the intervention, several instances arose where clinical staff required immediate knowledge regarding whether a patient was under the influence of substances and sought out research staff to request access to a rapid test cup, which the research staff readily provided. As the clinicians gained familiarity with the test cups, they gained confidence in the rapid test cup results and eventually allowed patients to use their research sample as their clinical sample. An additional complaint was that some clinicians noted that patients were consistently attending their research intervention appointments but skipping their clinical care appointments.

Research staff recorded over 50 interactions with patients involving spontaneous comments about the intervention in the research log. The majority of these comments were positive and related to patients’ perceived benefits of the intervention. Several patients reported that the intervention was helping them to maintain their sobriety. Four patients recounted specific instances in which they were experiencing a craving to use substances and thought about the incentives they hoped to earn to assist them in fighting the craving. In one of these cases, the participant stated while he was picking that he had really wanted to have a drink the previous weekend but that he did not want to have his picks reset. He then pulled out a $20 voucher and was so excited that he started to dance around the office. He stated:

“That reward was so worth not having a drink over the weekend!”

Several participants also reported that they had recommended participation in the intervention to other patients. Additional positive comments included appreciating being able to see their urine test cup results immediately and that the intervention gave them a documented record of urine screens that their clinicians could access. Patients also expressed satisfaction with the wide selection of items available at the VHA gift shop that could be purchased with their vouchers. The majority of the complaints about the intervention centered on disappointment with not drawing the larger vouchers.

## Discussion

The final step in conducting our process evaluation was to triangulate the results from the various data sources to answer the questions that were developed based on the elements of the selected frameworks (see Table 1) and to summarize lessons learned (see Table 6).

**Table 6:** Suggestions to enhance implementation efforts based on the RE-AIM framework

Reach	• Target intervention to patients that will be attending treatment at least twice per week for other treatment services.
Effectiveness	• Share results of VA and non-VA trials
Adoption	• Solicit explicit support from the highest levels of the organization through, for example, performance measures or treatment recommendations.
	• Identify or create measures of clinic effectiveness which can be utilized to identify gaps in performance and monitor the impact of implementation.
	• Solicit agreement in advance for designated funding.
	• Educate leadership about time commitment related to the intervention and potential strategies for integrating the intervention into current practices.
	• Adopt incrementally. Start with a specific treatment track or clinic to reduce staff and funding burden until local evidence of effectiveness and feasibility is available to support spread
Implementation	• Disseminate information to educate staff about the process, goals, and value/effectiveness of the intervention and engage them in planning for the intervention from the start.
	• Provide expert consultation on how to adapt the intervention for specific clinic environments.
	• Train staff on urine test cups and breathalyzer including sensitivity and specificity of the screen results.
	• Make scripts available for communicating positive and negative test results to patients.
	• Supply a tracking database to ensure consistency in awarding prize picks.
	• Provide a step by step intervention appointment protocol.
	• Facilitate documentation in the electronic health record.
Maintenance	• Ensure all staff is aware of their responsibilities related to incorporating information from the intervention into clinical interactions with patients to facilitate integration into the clinic.
	• Consider option of having case managers administer the intervention to their own patients rather than having one or two individuals responsible for the intervention.

### Reach

Screening data indicated that 60% of potential participants agreed to enroll in the intervention. The most common reasons for declining to participate related to the time commitment involved in having to attend appointments twice per week. This would suggest that the intervention may be most appropriately targeted to patients that will be coming to the clinic two or more days per week for other treatment services so as to reduce trips to the facility solely for the purpose of the 15 minute intervention appointment. African American patients and patients with stimulant dependence diagnoses agreed to participate at higher rates than patients in other demographic and diagnostic categories. The reliability of this observation as well as its clinical significance would need to be assessed in future research. It is clear from the patient survey and research log data that the vast majority of patients that agreed to participate felt the intervention experience was a positive one.

### Effectiveness

The primary outcomes of this study are reported elsewhere [19]. However, in brief, in the alcohol dependent subgroup, incentive participants submitted significantly more negative samples (13 vs. 11 samples), were retained significantly longer (7 vs. 6 weeks), and achieved significantly longer median durations of abstinence (16 vs. 9 consecutive visits compared to usual care participants).

Intervention effects were non-significant for the stimulant dependent subgroup. While these non-significant results for the stimulant dependent group were surprising, we believe these results were related to the characteristics of the recruited participants, specifically that they appeared to have less serious stimulant dependence compared to samples in several previous significant trials. Despite the non-significant results of this one trial, we believe that the extensive literature supporting incentive interventions with stimulant dependent patients supports implementation e.g., [33-36]; and further the results of this trial may provide some guidance in selecting patients most likely to benefit from incentive interventions.

### Adoption

Leadership identified some strong rationales for adoption of incentive programs. They perceived that incentive interventions would be valuable to improve their treatment retention performance ratings. Additionally, they believed national leadership viewed implementation of incentive interventions as a priority, and in fact, incentive interventions had been designated by national leadership as an evidence-based practice. The fact that the clinic leaders both expressed dissatisfaction with their own clinics’ performance on the treatment retention performance rating and perceived the incentive intervention as directly addressing this issue appeared to be the strongest motivator for adopting a change in practice. Clinic leaders that do not perceive issues with the effectiveness of their program at baseline may be less likely to express motivation to adopt practice change. This highlights the importance of having measures available to assess clinic effectiveness which can help to identify gaps in performance and assess the impact of changes in clinic practices.

However, these strong rationales for adoption could not override the greatest barrier, which was lack of resources in the form of staff time and funding for vouchers and urine testing supplies. Multiple data sources, including the ORCA, staff interviews, leadership interviews, and the research log, all indicated that lack of resources was the primary perceived barrier to adoption of the intervention. To address the perceptions regarding staff time, we can suggest two implementation strategies that may decrease leadership concerns about staffing burden: providing education in advance as regards the amount of time necessary to administer the incentive intervention (15 minutes or less per instance); and presenting various options for integrating the intervention into current clinic staffing patterns especially when group sessions are the predominant modality. While cost of the intervention is traditionally considered to fall under the *Implementation* construct of the RE-AIM framework, we consider cost under the *Adoption* construct given the high relevance of funding concerns to the decision whether or not to adopt this particular intervention. The mean total cost of the eight week intervention was $266.93 – $316.21 per patient depending on the salary rate of the individual administering the intervention. This cost could be reduced by using lower wage staffing. While a full cost-effectiveness evaluation is beyond the scope of this paper, the incentive intervention was shown to increase participants’ longest duration of abstinence during treatment [19] which is one of the best predictors of long-term patient outcomes [37-39]. Olmstead and colleagues have published two articles reporting on the cost-effectiveness of incentive interventions [40,41]. The potential for decreased societal costs related to medical expenses, criminal/legal costs, and lost productivity would suggest that this is a low price for an intervention that improves substance use disorders treatment outcomes. Providing education regarding the low cost and the cost-effectiveness of the intervention and soliciting agreement in advance from facility leadership for dedicated funding appear to be essential to adoption.

It should be noted that adoption of many new evidence-based practices into an existing clinic milieu will require additional resources, so such barriers are not unique to incentive interventions. Another potential implementation strategy may be to assist clinic leadership in identifying current clinic practices that are not evidence-based or that have been shown to contribute little to treatment outcomes, e.g., didactic education [42]. The implementation team and the clinic leadership could then focus on strategies for reallocating existing resources from current non-evidence based practices to implementation of evidence based practices not currently available in the clinic.

Clinic leadership also indicated that it would be an easier “sell” to facility leadership to begin by targeting the intervention to a small group of patients. This would offer a way to test the intervention in the clinic and collect local outcome data that could then be used to advocate for sustained funding support for the intervention at a later date. Starting with a “test” of the intervention could also lessen staff resistance and allow them to observe the effectiveness of the intervention in their own clinic and the impact of the intervention on their workload prior to committing to sustained implementation.

### Implementation

Once a clinic has made a decision to adopt the incentive intervention, supports must be in place to ensure that implementation is carried out with high levels of fidelity and consistency. The staff and leadership interviews suggested several ways that high quality implementation could be enhanced. These include: 1) involving staff in implementation planning to increase knowledge regarding essential intervention elements; 2) providing support for adapting the intervention to fit the current clinic structure through expert consultation and recommended strategies based on common clinic structures; and 3) defining clear roles for all clinical staff related to the intervention so that it would be a clinic-wide initiative rather than viewed as only the responsibility of specific people. In addition to these supports, the research log suggested that it would be beneficial to train all staff on the use of rapid urine test cups and their specificity and sensitivity prior to implementation to address concerns about the reliability of that technology.

Other observations related to implementation tools that could be used to enhance consistent delivery of this intervention come from our experience in training the research staff. Research assistants (RAs) were provided with training on the accurate use of the rapid urine test cups and the breathalyzer unit and with scripts for how to communicate screening results to patients. Additionally, a database was created that tracked the number of picks that a patient was entitled to at a given appointment based on their previous record of attendance and test results. This simplified the work of the RAs and ensured the consistency with which the incentives were distributed. RAs were also provided with a manual that described the steps of an intervention appointment from beginning to end. Once an RA had completed their training and read the manual, they completed a mock intervention appointment with one of the investigators (HH, DK) prior to completing intervention appointments with patients. These same training tools and steps could be provided to clinical staff to ensure consistent delivery of the intervention.

### Maintenance

In examining *Maintenance,* we were primarily interested in adaptations that could be made to the incentive intervention that would enhance the probability that the intervention would be sustained. As reflected in the barriers to adoption, dedicated staff time and funding would be essential to maintenance and should be secured prior to a decision to adopt the intervention. In addition, both staff and leadership interviews indicated that fully integrating the intervention into the clinic would be essential to maintenance. The intervention, as delivered during the study, was viewed as a stand-alone intervention that was the responsibility of specific individuals (in this case, research staff). While some individual staff members did report utilizing information about patients’ progress in the intervention in their therapeutic interactions, the patient survey information would suggest that this was infrequent. It does not seem that clinicians were uninterested or opposed to being more involved, as staff at both clinics provided unsolicited suggestions regarding ways that clinical staff could be kept more up-to-date on patient progress in the intervention. Therefore, it is more likely that they did not know enough about the intervention or how best to incorporate information from the intervention into their therapeutic interactions. This could be mitigated by providing better education to all staff about the intervention and by developing explicit expectations regarding how staff should integrate information from the intervention into their interactions with patients. Another option would be to have each case manager provide the incentive intervention to their case load rather than assigning administration of the incentive intervention to one or two isolated individuals. This option would also eliminate the staff concern that patients were attending their intervention appointments more regularly then their clinical appointments as these would be provided concurrently.

### Evidence

Ratings on the ORCA indicated that staff at both facilities generally agreed with the evidence supporting the intervention, that the intervention met the needs of their patients, and that the intervention fit with their treatment philosophy. Staff interviews supported results from the ORCA. The research log data also supported staff’s reported enthusiasm for the intervention with multiple reports of clinical staff members assisting research staff with recruitment and facilitating communication between research staff and study participants.

However, despite this general enthusiasm, there were clear exceptions to this support with a small minority of staff reporting a serious conflict between their personal treatment philosophy and incentive interventions. They strongly felt that the incentives created a “false”, external motivation for abstinence that would undermine patients’ internal motivation for change and would interfere with longer term treatment success. It is likely that these beliefs are even more prevalent in community treatment settings that do not have the strong focus on evidence-based practice currently promoted within the VHA system. While these beliefs tend to be highly entrenched, having discussions up front with staff and leadership to identify such beliefs or concerns and to determine what types of evidence staff would find compelling might encourage the staff to consider supporting implementation. For example, staff may simply not be aware of the level and detail of evidence supporting incentive interventions. They also may benefit from a more in-depth understanding of how behavioral reinforcements function in a variety of settings. They may be encouraged by discussing incentive interventions with other clinics that have already implemented or, as suggested earlier, they may be willing to engage in a brief test of incentive interventions in their own clinic that would allow staff to see the benefits and risks of the intervention among their own patients. The important point is to engage the staff in a respectful discussion about implementation early on so that their concerns can be heard and addressed prior to a formal decision to make a change in how they are going to treat their patients. The Promoting Awareness of Motivational Incentives (PAMI) website, based on knowledge gained from the National Institute of Drug Abuse (NIDA) Clinical Trials Network (CTN) Motivational Incentives for Enhanced Drug Abuse Recovery study, provides several excellent resources for staff education and training including testimonial videos from patients and clinicians regarding the impact of incentive interventions on clinic culture and patient outcomes (http://www.bettertxoutcomes.org/bettertxoutcomes/PAMI.html).

### Context

ORCA survey results indicated that staff perceived a generally positive leadership/staff culture and positive leadership practices. The ORCA survey supported interview data, indicating that staff generally disagreed that they had access to the necessary resources to support innovation.

Despite general agreement with the evidence supporting incentive interventions and contexts that support innovation in general, the clinics involved in this study perceived substantial barriers to implementation. The implication is that barriers may be perceived as even more substantial in sites that do not have these supportive characteristics. This highlights the importance of conducting local assessment of *Evidence* and *Context* when undertaking implementation efforts [43].

#### Limitations

One limitation of this study is that the ORCA was only collected after the intervention had been running in the clinic for 18 months. It would have been ideal to have also collected this information prior to the start of the intervention period, as experiencing the intervention functioning in their clinic may have altered staff’s attitudes toward the intervention. Previous studies have indicated that providers who have prior experience with tangible incentive programs display more positive beliefs about incentive programs and lower ratings of barriers to adoption than those who report no experience with such programs [44-46]. These findings reinforce the suggestion, also made by other authors, that exposing treatment providers to incentive programs through a pilot or targeted demonstration may be a useful strategy for encouraging dissemination [47]. A second limitation was that the sample size for the staff interviews was small, particularly in site two, and therefore may not adequately represent the opinions of the population of clinic staff at the sites. Clinic staff that agreed to participate in the interviews may have had more positive opinions about the intervention compared to those that declined to be interviewed. Finally, because this was an effectiveness trial with research staff conducting the incentive intervention rather than an implementation trial with the clinical staff conducting the intervention, staff perception of the intervention, particularly related to its impact on workload, may be quite different than if they were responsible for conducting the intervention themselves.

## Conclusion

An implementation-focused process evaluation guided by the RE-AIM and PARIHS frameworks led to significant insights about the processes involved in implementation of incentive interventions and allowed us to formulate concrete suggestions about implementation strategies. Some of the most important insights were: 1) the need to spend substantial effort on education and persuasion strategies in sites where staff are skeptical of the value of implementing incentive programs, and 2) the need to assist leadership in developing strategies for financing and staffing. Overall, the study demonstrates the value of conducting implementation process evaluations as a concurrent part of effectiveness research.

The goals of this paper were to describe an implementation-focused process evaluation conducted during a randomized trial and distil findings into practical guidance to others who wish to implement incentive interventions in substance use disorders clinics. Table 6 presents a summary of the key lessons learned from this effort. It is our hope that this summary speaks to the value of the effort expended on the process evaluation and highlights issues to consider when attempting implementation of an incentive intervention.

## Competing interests

The authors declare that they have no competing interests.

## Authors’ contributions

HH conceived of the study, led the study design, interpreted data and drafted the manuscript. CS participated in the study design, provided extensive consultation on data interpretation, and provided critical revisions to the manuscript. AB developed the data collection strategies, provided database management, produced the demographic table and recruitment figure, and provided comments on the manuscript. SN participated in study design, performed statistical analyses, and provided comments on the manuscript. MS participated in study design and provided critical revisions to the manuscript. DK participated in study design and provided critical revisions to the manuscript. All authors read and approved the final manuscript.
